# Supplementary material for: Canadian Collaboration to Identify a Minimum Dataset for Antimicrobial Use Surveillance for Policy and Intervention Development across Food Animal Sectors
Source: Antibiotics (Basel). 2022 Feb 10;11(2):226. doi: 10.3390/antibiotics11020226 (PMC8868246; doi:10.3390/antibiotics11020226)
Supplement: Supplementary file 1 [file antibiotics-11-00226-s001.zip › antibiotics-1569050-supplementary.pdf]

Supplementary Materials

Table S1. Affiliations of the working group involved in the development of the minimum dataset for antimicrobial use surveillance (MDS-AMU-surv) between 2016 and 2017

**Organization**	**Type**
Agriculture and Agri-food Canada	Federal government
Animal Nutrition Association of Canada	Industry
Canadian Animal Health Institute	Industry
Canadian Aquaculture Industry Alliance	Industry
Canadian Food Inspection Agency	Federal government*
Canadian Hatching Egg Producers	Industry
Canadian Pork Council	Industry
Canadian Poultry and Egg Processors Council and the Canadian Hatcheries Federation	Industry
Canadian Sheep Federation	Industry
Canadian Veterinary Medical Association	Professional association
Chicken Farmers of Canada	Industry
Council of Chief Veterinary Officers (Antimicrobial use task team)	Federal-provincial-territorial task team
Dairy Farmers of Canada	Industry
Dairy Farmers of Ontario	Industry
Egg Farmers of Canada	Industry
Le Centre de développement du porc du Québec	Industry
National Cattle Feeders Association	Industry
Ontario Ministry of Agriculture, Food and Rural Affairs	Provincial government*
Public Health Agency of Canada	Federal government*
TDV Global	Private
Turkey Farmers of Canada	Industry
University of Calgary	Academia
Veal Farmers of Ontario	Indutry
Veterinary Drugs Directorate – Health Canda	Federal government

*Lead coordinating agencies/government for the MDS-AMU-surv

Table S2. Questionnaire, Inclusion and Feasibility Score

[1] **Inclusion Scores**: **0**=Not required; **1**=Must be included; **2**=Nice to have.
[2] **Feasibility Scores**: **0**=Not feasible; **1**=Currently being collected by your org.; **2**=Could start collecting with minimal effort; **3**=Could start collecting with significant effort.

1.00	**Respondent Information**	
1.10		Name of respondent
**1.20**		**Organization represented**

2.00	**At what level will these data be collected?** Please select only one from the list below by entering "1".	
2.10		Pharmaceutical manufacturer sales data (e.g. CAHI - legislative change will make reporting mandatory)
2.20		Veterinary prescription data
2.30		Veterinary drug sales data
2.40		Veterinary records (real time or sample of 48 hour diary data)
2.50		Farm level antimicrobial use data
2.60		Other - Levels
2.61		Specify *Other*:
Section	**OUTPUTS**	**DATA REQUIRE TO PROVIDE OUTPUT**
3.00	**A list of active antimicrobial ingredient (AI) used (Yes/No)**	
3.10		Product ID
3.20		Active Ingredient(s)
4.00	**Counts (frequencies) of AI Use**	
4.10		Count of sales by pharmaceutical manufacturers
4.20		Count of purchases by pharmacies
4.30		Count of purchases by veterinary clinics
4.40		Count of prescriptions
4.50		Count of purchases by farms
4.60		Count of medicated/non-medicated rations
4.70		Count of treatments
4.80		Other - Counts
4.81		Specify *Other*:
5.00	**Quantity of AI Use ("per" the denominator selected below)**	
5.10		Mgs distributed (Pharmaceutical Manufacturers)
5.20		Mgs purchased (Vet./Pharm.)
5.30		Mgs used (may require data from Section 5)
5.40		Mgs sold by pharmaceutical manufacturers
5.50		Mgs purchased by pharmacies
5.60		Mgs purchased by veterinary clinics
5.70		Mgs purchased by farms
5.80		Mgs prescribed
5.90		Other - Quantities
5.91		Specify *Other*:
6.00		**Prescribing/dispensing/use details** (Data in **Section 6** are required for Quantitative outputs)
6.10		Production class or phase
6.20		Route of administration
6.30		Injection - Dose
6.31		Injection - Frequency of administration
6.32		Injection - Duration of treatment

6.40		Feed - Dose (e.g. grams/tonne)
6.41		Feed intake (Ration starting weight or age and days fed)
6.50		Water - Dose (e.g., grams/liter)
6.51		Water - Dose (Concentration of drug per product)
6.52		Water intake (Starting weight or age and days exposed)
6.60		Count (%) of treated animals
6.70		Other prescription information
6.71		Specify other:
7.00	**Denominator**	
7.10		Number of animals exposed
7.20		Number of animals not exposed
7.30		Number of animals processed
7.40		Time: Average days/weeks/months in each production phase, e.g., Avg. days in grower-finisher phase
7.50		Location: Province/region, sentinel site area…
7.60		Other Denominators
7.61		Specify Other:
9.00	**Reason for use**	
9.10		Primary reason (Prevention/Treatment)
9.20		Secondary reasons (Commodity specific disease categories)
9.30		Target pathogen, e.g., specific bacteria
9.40		Expert opinion to establish reasons for use of an antimicrobial
9.50		Other Reasons
9.51		Specify Other:
10.00	**Other data of interest**	
10.10		Extra-label drug use (ELDU Yes/No)
10.20		Imported (DIN/Non-DIN Yes/No)
10.30		Compounded (Yes/No)
10.40		Emergency drug release (EDR Yes/No)
10.50		Economic indicators - production parameters (*from discusion on Outcomes*)
10.60		Animal health indicators
10.70		Other Data of Interest
10.71		Specify Other:
**11.00**	**Report by**	
11.10		Active antimicrobial ingredient
11.11		Antimicrobial class
11.20		Categories of importance to human medicine (VDD-HC Cat. I, II, III and IV)
11.30		Animal species
11.40		Commodity/sector
11.50		Farm demographics (e.g., Production type)
11.60		Veterinarian demographics (e.g. specialization)
11.70		Temporal trends (Years, months…)
11.80		Spatial trends (Canadian Provinces/Regions)
11.90		Other items to report by
11.91		Specify *Other*:
**12.00**	**Sample size**	
12.10		Census
12.30		A proportion of the population that would provide a valid and representative sample, mindful of cost and effort
12.40		Other sample size considerations
12.41		Specify *Other*:
**13.00**	**Who would collect/submit these data?**	
13.10		Commodity organizations
13.20		Veterinarians
13.30		Pharmaceutical industry

13.40		Provincial gov. agency
13.50		Federal gov. agency
13.60		Other Orgs
13.61		Specify *Other*:
**14.00**	**Short summary: What is your organization doing currently re: AMU data collection and/or future plans**	
14.10		
